# Neutrophil‐to‐lymphocyte ratio as a predictor of clinical outcomes in critically ill COVID‐19 patients: A retrospective observational study

**DOI:** 10.1002/hsr2.844

**Published:** 2022-09-15

**Authors:** Husain S. Ali, Dore C. Ananthegowda, Ebrahim M. A. Ebrahim, Nevin Kannappilly, Mohammad Al Wraidat, Ahmed S. Mohamed, Mohamad Y. Khatib

**Affiliations:** ^1^ Department of Medical ICU/Medicine Hamad General Hospital Doha Qatar; ^2^ Intensive Care Unit, Hazm Mebaireek General Hospital Doha Qatar; ^3^ Department of Medical Education Hamad General Hospital Doha Qatar

**Keywords:** COVID‐19, ICU mortality, mechanical ventilation, neutrophil‐to‐lymphocyte ratio, SARS‐CoV‐2

## Abstract

**Background:**

Timely identification of patients at risk of worse clinical outcomes is vital in managing coronavirus disease 2019 (COVID‐19). The neutrophil‐to‐lymphocyte ratio (NLR) calculated from complete blood count can predict the degree of systemic inflammation and guide therapy accordingly. Hence, we did a study to investigate the role of NLR value on intensive care unit (ICU) admission in predicting clinical outcomes of critically ill COVID‐19 patients.

**Methods:**

We conducted a retrospective analysis of electronic health records of COVID‐19 patients admitted to ICUs at Hazm Mebaireek General Hospital, Qatar, from March 7, 2020 to July 18, 2020. Patients with an NLR equal to or higher than the cut‐off value derived from the receiver operating characteristic curve were compared to those with an NLR value below the cut‐off. The primary outcome studied was all‐cause ICU mortality. The secondary outcomes evaluated were the requirement of mechanical ventilation and ICU length of stay (LOS).

**Results:**

Five hundred and nineteen patients were admitted to ICUs with severe COVID‐19 infection during the study period. Overall, ICU mortality in the study population was 14.6% (76/519). NLR on ICU admission of ≥6.55 was obtained using Youden's index to predict ICU mortality, with a sensitivity of 81% and specificity of 41%. Mortality was significantly higher in patients with age ≥60 years (*p* < 0.001), chronic kidney disease (*p* = 0.03), malignancy (*p* < 0.002), and NLR ≥ 6.55 (*p* < 0.003). There was also a significant association between the requirement of mechanical ventilation (34.7% vs. 51.8%, *p* < 0.001) and increased ICU LOS (8 vs. 10 days, *p* < 0.01) in patients with ICU admission NLR ≥ 6.55.

**Conclusion:**

Higher NLR values on ICU admission are associated with worse clinical outcomes in critically ill COVID‐19 patients.

## 1 INTRODUCTION

Complete blood count (CBC) is a simple, low‐risk, inexpensive test routinely requested in clinical practice. Despite being used for many years, new implications of CBC are still being explored, with neutrophil‐to‐lymphocyte ratio (NLR) being one of them. NLR is calculated from the white cell differential count as a ratio of the number of neutrophils divided by the number of lymphocytes. It is an inflammatory biomarker used in the early detection of sepsis,^1^ screening and diagnosis of inflammatory diseases,^2^ identifying and managing surgical emergencies,^3^ predicting postoperative complications,^4^ and prognostication of malignancies.^5^

Coronavirus disease (COVID‐19) is an infectious respiratory system illness caused by severe acute respiratory syndrome coronavirus 2 (SARS‐CoV‐2). Initial cases were identified in Wuhan city, China, in December 2019. Since then, the infection quickly spread and was declared a pandemic by World Health Organization (WHO) on March 11, 2020.^6^ The majority of infected people experience mild to moderate symptoms and recover without specific treatment. However, older people and those with medical comorbidities are more likely to develop serious illnesses. So far, more than 599 million cases and 6.4 million deaths have been reported globally due to COVID‐19.^7^

There is growing evidence that a hyperinflammatory response to SARS‐CoV‐2 results in rapid deterioration and worse outcomes in critically ill COVID‐19 patients.^8^ However, a quick, easy‐to‐perform, and reliable test to detect pathogenic inflammation and guide management before clinical worsening occurs is still lacking. NLR can be used by itself or in combination with other criteria to identify patients at risk of disease progression and intervene promptly. However, the value of NLR associated with severe disease and adverse outcomes is not well established. This persuaded us to conduct a retrospective study investigating the role of NLR as a marker to identify high‐risk patients and predict unfavorable clinical outcomes in critically ill COVID‐19 patients.

## 2 MATERIALS AND METHODS

### 2.1 Study design, participants, and setting

We retrospectively analyzed electronic health record data of patients admitted to intensive care units (ICUs) at Hazm Mebaireek General Hospital, Qatar, between March 7, 2020 and July 18, 2020. Medical Research Center (MRC) at Hamad Medical Corporation, Qatar, validated the study protocol and waived the need for informed consent (protocol no.: MRC‐01‐20‐1155). The inclusion criteria were: COVID‐19 positivity established by reverse transcription polymerase chain reaction of nasopharyngeal swabs, age more than 18 years, and ICU admission for more than 24h.

### 2.2 Data collection

Data collected were demographic characteristics, comorbid conditions, blood test results, need for mechanical ventilation, ICU length of stay (LOS), and mortality. The NLR for each patient was calculated by dividing the absolute neutrophil count (ANC) by the absolute lymphocyte count (ALC) obtained from the CBC test. The normal adult range of ANC was 2000–7000 per microliter, and for ALC, it was 1000–3000 per microliter. The primary outcome investigated was all‐cause ICU mortality in relation to NLR. The secondary outcomes evaluated were the requirement for mechanical ventilation and ICU LOS.

### 2.3 Statistical analysis

The IBM Statistical Package for Social Sciences (SPSS version 24.0) was used to analyze the data. Numerical variables were summarized using mean ± SD or median (IQR) according to distribution, while categorical variables were summarized as frequencies or percentages. The *χ* ^2^ test and Fisher's exact test were used to compare categorical data. As the numerical data followed a non‐normal distribution, two‐tailed nonparametric tests, namely, Spearman's correlation and Mann–Whitney *U*‐test, were used for correlation analysis and comparison of mean ranks, respectively. The area under receiver‐operating characteristic curve was calculated to identify prognostic cut‐off points for mortality. Based on the best‐identified cut‐off point, NLR was dichotomized into two categories. Outcome predictors were obtained by multiple logistic regression (LR), which also controls for possible confounding bias. Alpha was set at 0.05 to determine statistical significance.

## 3 RESULTS

### 3.1 Patient characteristics

During the study period, 519 patients with COVID‐19 infection were admitted to ICUs at Hazm Mebaireek General Hospital. The mean age of patients was 51.6 ± 13.6 (mean ± SD) years, and 93.3% (484/519) of them were males. The most frequent comorbid conditions were diabetes mellitus (259/519, 49.9%) and hypertension (231/519, 44.5%). Table 1 summarizes the study population's sociodemographic characteristics and pre‐existing comorbidities.

**Table 1:** Patient characteristics

Variable	Total (*n* = 519)	Outcome		*χ* ^2^	*p* Value
		Survived	Died		
Age					
<60 years	368	346 (94%)	22 (6%)	76	<0.001
≥60 years	151	97 (64.2%)	54 (35.8%)		
Gender					
Male	484 (93.3%)	413 (85.3%)	71 (14.7%)	0.004	0.95
Female	35 (6.7%)	30 (85.7%)	5 (14.3%)		
Comorbid conditions^a^					
Diabetes mellitus	259	215 (83%)	44 (17%)	2.27	0.13
Hypertension	231	183 (79.2%)	48 (20.8%)	12.5	<0.001
Dyslipidemia	60	50 (83.3%)	10 (16.7%)	0.22	0.64
Cardiovascular disease	76	59 (77.6%)	17 (22.4%)	4.25	0.04
Chronic kidney disease	57	37 (64.9%)	20 (35.1%)	21.4	<0.001
Respiratory disease	36	28 (77.8%)	8 (22.2%)	1.8	0.18
Chronic liver disease	7	4 (57.1%)	3 (42.9%)	4.52^*^	0.03
Malignancy	14	7 (50%)	7 (50%)	14.4	<0.001
NLR at ICU admission^b^					
<6.55	193	178 (92.2%)	15 (7.8%)	11.6	<0.001
≥6.55	326	265 (81.3%)	61 (18.7%)		

Variable	Total (*n* = 519)	Nonintubated	Intubated	*χ* ^2^	*p* Value
NLR at ICU admission^b^					
<6.55	193	126 (65.3%)	67 (34.7%)	14.34	<0.001
≥6.55	326	157 (48.2%)	169 (51.8%)		

*Note*: *N* = 519, output of *χ* ^2^ or *Fisher's exact tests. *p* < 0.05 is statistically significant.

Abbreviations: ICU, intensive care unit; NLR, neutrophil‐to‐lymphocyte ratio.

^a^ Compared to not having the comorbid conditions.

^b^ Percentage within the group.

### 3.2 Outcomes

**Figure 1:**
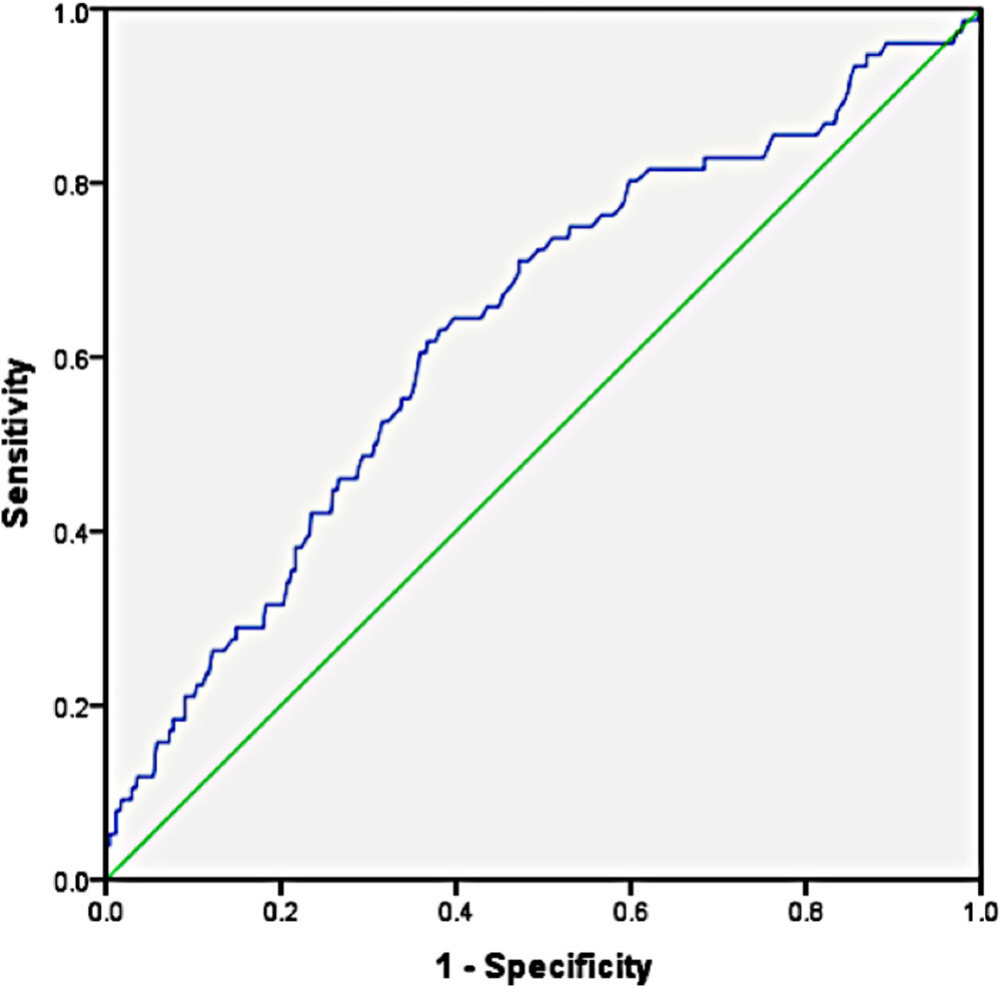
Receiver‐operating characteristic curve illustrating the diagnostic ability of intensive care unit admission NLR in critically ill coronavirus disease 2019 patients. The areas under the curve were calculated as 0.636 (95% confidence interval: 0.57–0.71). NLR cutoff point predicting mortality was 6.55 based on Youden's index with a sensitivity of 81% and a specificity of 41%. NLR, neutrophil‐to‐lymphocyte ratio.

The all‐cause mortality for COVID‐19 patients admitted to ICUs was 14.6% (76/519). NLR cut‐off value predicting mortality, obtained using Youden's index, was 6.55 for NLR on ICU admission, with a sensitivity of 81% and specificity of 41% (Figure 1). Several variables were evaluated for association with mortality, as presented in Table 1. Mortality was significantly higher in age group ≥60 years (*p* < 0.001), in patients with pre‐existing hypertension (*p* < 0.001), cardiovascular disease (*p* = 0.04), chronic kidney disease (*p* < 0.001), chronic liver disease (*p* = 0.03), malignancy (*p* < 0.001), and NLR ≥ 6.55 on ICU admission. Variables with *p* ≤ 0.25 were entered into the LR model using the forward LR method to eliminate confounders and insignificant factors (Table 2).

**Table 2:** Predictors of mortality

Variables	*B*	SE	Wald	AOR	95% CI	*p* Value
Age ≥60 years	1.95	0.295	43.89	7.0	3.94–12.45	<0.001
Chronic kidney disease	0.78	0.35	4.84	2.2	1.09–4.36	0.03
Malignancy	1.84	0.59	9.55	6.3	1.96–20.31	0.002
ICU admission NLR ≥ 6.55	1.01	0.34	9.1	2.8	1.43–5.32	0.003

*Note*: Forward logistic regression method. Reference categories: age < 60 years, no chronic kidney disease, no malignancy, ICU admission NLR < 6.55. *p* < 0.05 is statistically significant.

Abbreviations: AOR, adjusted odds ratio; CI, confidence interval; ICU, intensive care unit; NLR, neutrophil‐to‐lymphocyte ratio.

Overall, 45.5% (236/519) of the study population required intubation, and the need for mechanical ventilation was significantly higher in patients with NLR ≥ 6.55 on ICU admission, as shown in Table 1.

NLR median values on hospital and ICU admission were significantly higher among patients who died or required mechanical ventilation (Table 3). A statistically significant correlation was noted between NLR on ICU admission and ICU LOS with Spearman's correlation coefficient (*R*s) = 0.157 (*p* < 0.001). Similarly, there was a weak positive correlation between NLR on hospital admission and hospital LOS with *R*s = 0.092 (*p* = 0.03).

**Table 3:** Median NLR values among different outcome groups

Parameters	Median (IQR)		*U*	*Z*	*r* Value	*p* Value
	Alive	Dead				
NLR on hospital admission	4.2 (3.9)	5.2 (4.8)	19,694.5	−2.37	0.104	0.02
NLR on ICU admission	7.8 (7.8)	11.1 (10.9)	21,407	−3.79	0.166	<0.001

Parameters	Nonintubated	Intubated	*U*	*Z*	*r* Value	*p* Value
NLR on hospital admission	3.8 (3.6)	4.8 (4.6)	39,173.5	−3.4	0.149	0.001
NLR on ICU admission	7.25 (6.8)	9.9 (9.74)	41,733.5	−4.91	0.215	<0.001

Parameters	NLR < 6.55	NLR ≥ 6.55	*U*	*Z*	*r* Value	*p* Value
ICU LOS (days)	8 (10)	10 (13)	36,774	−3.22	0.142	0.001

*Note*: Output of Mann–Whitney test comparing mean ranks. *U* and *Z*: test statistics. *r*: estimate of effect size indicating in small effect size <0.3 (Cohen, J., 1988. Statistical power analysis for the behavioral sciences. Lawrence Earlbaum Associates).^9^

Abbreviations: ICU, intensive care unit; IQR, interquartile range; LOS, length of stay; NLR, neutrophil‐to‐lymphocyte ratio.

## 4 DISCUSSION

Our single‐center retrospective study revealed a significant association of NLR value on ICU admission with ICU mortality, need for mechanical ventilation, and ICU LOS, with higher NLR values predicting worse outcomes.

NLR reflects the balance between innate (neutrophil granulocytes) and adaptive (lymphocytes) immune responses. The rise in neutrophil count and decline in lymphocyte count is a multifactorial dynamic process depending on finetuning and regulation of various immunologic, neuroendocrine, humoral, and biologic processes such as margination/demargination, mobilization/redistribution, accelerated/delayed apoptosis, the influence of stress hormones, and sympathetic/parasympathetic imbalances.^10^ Clinicians have been using inflammatory markers such as C‐reactive protein (CRP), lactate dehydrogenase, ferritin, and interleukin (IL) levels to assess disease severity, predict outcomes, and guide therapy in critically ill COVID‐19 patients. However, some of these tests might not be easily available, especially in remote areas, to make clinical decisions. A recent study has revealed strong correlation between NLR and CRP values (*r* = 0.738, *p* < 0.001) in COVID‐19 cases.^11^ Hence, NLR can be used as a rapid, easy, and economical test to predict the severity of inflammation and identify high‐risk patients who need close monitoring and aggressive therapy.

The physiological values of NLR in a healthy adult population depend on ethnicity and other factors like smoking, obesity, and diabetes mellitus. The average value of NLR observed in Afro‐American, Hispanic, and Caucasian individuals was 1.76, 2.08, and 2.24, respectively.^12^ A study analyzing hemograms of the Asian population revealed a mean NLR value of 1.65 in healthy Korean adults.^13^ There is no consensus on the cut‐off value to determine normal and elevated NLR, especially in COVID‐19 patients. In agreement with our results, a recent meta‐analysis of 38 studies showed that higher NLR values on admission were associated with increased risk of severity and mortality in COVID‐19, suggesting that this readily available biomarker can predict the prognosis of COVID‐19 patients.^14^ In determining the optimal cut‐off value of NLR, two previous studies used 7.9 (AU: 0.8; sensitivity: 65.3%; specificity: 90.6%) and 11.8 (AUC: 0.9, sensitivity: 97.5%; specificity: 78.1%) to predict mortality.^15^, ^16^ Another study identified NLR value above 4.94 was associated with intubation at admission (*p* < 0.02), longer mean ICU stay (2.6 vs. 1.1 days; *p* < 0.015) and more days utilizing mechanical ventilation (2.2 vs. 0.9 days; *p* < 0.019).^17^

Our study has some limitations. First, we exclusively studied NLR values in critically ill COVID‐19 cases, which is a tiny proportion of the total number of infected cases. Hence, our sample size was small, and also, we could not evaluate the relationship between NLR values and noncritical COVID‐19 patients. Second, due to the retrospective study design, we could not exclude the impact of treatments received before ICU admission on the outcome, which may have resulted in a potential overestimation of the NLR performance (e.g., the NLR may be affected by steroid therapy). Third, we did not have pre‐existing data on the average NLR values in the healthy local population compared with COVID‐19 cases. Finally, although the study was performed in a tertiary care COVID facility, it is a single‐center study among the predominantly male population (93.3%), thereby limiting its generalizability.

## 5 CONCLUSION

This retrospective single‐center study of 519 critically ill COVID‐19 patients revealed that the NLR value ≥6.55 on ICU admission was associated with higher ICU mortality and the need for mechanical ventilation. Despite the limitations, our results are in line with the previously demonstrated predictive ability of NLR and conducted in a previously unstudied patient population. Therefore, we believe that the trends and predictors shown here are likely real while realizing that external validation would strengthen our conclusions.

## AUTHOR CONTRIBUTIONS

**Husain S. Ali**: Conceptualization; methodology; writing – original draft; writing – review and editing. **Dore C. Ananthegowda**: Conceptualization; methodology. **Ebrahim M. A. Ebrahim**: Formal analysis; investigation. **Nevin Kannappilly**: Data curation; investigation. **Mohammad Al Wraidat**: Data curation; investigation. **Ahmed S. Mohamed**: Conceptualization; project administration. **Mohamad Y. Khatib**: Supervision; writing – review and editing.

## CONFLICT OF INTEREST

The authors declare no conflict of interest.

6

## ETHICS STATEMENT

The study was approved by the MRC at Hamad Medical Corporation (protocol no.: MRC‐01‐20‐1155). The research was conducted per the ethical standards noted in the 1964 Declaration of Helsinki and its later amendments or comparable ethical standards. No consent was obtained due to the retrospective nature of the study.

## TRANSPARENCY STATEMENT

The lead author Husain Shabbir Ali affirms that this manuscript is an honest, accurate, and transparent account of the study being reported; that no important aspects of the study have been omitted; and that any discrepancies from the study as planned (and, if relevant, registered) have been explained.

## Data Availability

The data supporting this study's findings are available from the corresponding author upon reasonable request.
